# Morphological evaluation of incisive canal between dentate and maxillary central incisor loss subjects in a Vietnamese population using cone beam computed tomography: a retrospective cross-sectional study

**DOI:** 10.3389/fdmed.2026.1833001

**Published:** 2026-06-17

**Authors:** Anh Tuan Dang, Hai Thanh Pham, Elena Saraí Baena-Santillán, José de Jesús Navarrete-Hernández, Carlos Enrique Cuevas-Suárez, Rim Bourgi

**Affiliations:** 1 Faculty of Dentistry, Haiphong University of Medicine and Pharmacy, Haiphong, Vietnam; 2 Department of Odonto-stomatology, Haiphong Medical University Hospital, Haiphong, Vietnam; 3 Dental Materials Laboratory, Academic Area of Dentistry, Autonomous University of Hidalgo State, San Agustín Tlaxiaca, Mexico; 4 Benemerita Universidad Autonoma de Puebla, Puebla, Mexico; 5 Department of Biomaterials and Bioengineering, INSERM UMR_S 1121, University of Strasbourg, Strasbourg, France; 6 Department of Restorative Dentistry, School of Dentistry, Saint-Joseph University of Beirut, Beirut, Lebanon; 7 Department of Restorative Sciences, Faculty of Dentistry, Beirut Arab University, Beirut, Lebanon

**Keywords:** alveolar bone resorption, cone-beam computed tomography, incisive canal, maxillary central incisor, morphologic evaluation

## Abstract

Accurate assessment of the incisive canal (IC) is crucial for planning dental implant procedures in the aesthetic anterior maxilla, yet population-specific data on its morphology, particularly following maxillary central incisor loss (MCIL), remains limited.

**Objective:**

This study aimed to evaluate and compare the three-dimensional morphology of the IC and the buccal alveolar bone plate (BABP) between dentate subjects and those with MCIL in a Vietnamese population using cone-beam computed tomography (CBCT).

**Methods:**

This retrospective cross-sectional study analyzed 138 CBCT scans, divided into a Dentate group (*n* = 100) and an MCIL group (*n* = 38). Morphological classification of the IC was performed on sagittal, coronal, and axial planes. Quantitative linear and areal measurements of the IC (length, diameters, area, angulation) and the adjacent BABP (width at crestal, middle, and nasal levels; height) were obtained. Inter-group and gender-based comparisons were conducted using independent t-tests and Chi-square tests, with age adjustment via analysis of variance (ANCOVA) for continuous variables.

**Results:**

Gender-based analysis revealed significant sexual dimorphism, with males exhibiting larger IC diameters and greater BABP widths compared to females (*p* < 0.05). No statistically significant differences were found in the morphological shape, division pattern, or any quantitative parameter (including sagittal length) of the IC between the Dentate and MCIL groups (*p* > 0.05). In contrast, all width dimensions of the BABP were significantly reduced in the MCIL group, most notably at the crest (4.50 ± 1.50 mm vs. 7.36 ± 1.56 mm in Dentate, *p* < 0.00001).

**Conclusion:**

The loss of maxillary central incisors is associated with significant horizontal resorption of the BABP but does not significantly alter the morphology or dimensions of the incisive canal.

## 1 Introduction

The maxillary incisive canal (IC) is a critical anatomical structure in the anterior maxilla, which houses the nasopalatine neurovascular bundle and is intimately related to the apices and palatal surfaces of the maxillary central incisors (1, 2). Its location, posterior to the maxillary central incisor roots and encased in dense cortical bone, renders it a vital consideration in a multitude of dental procedures, from orthodontic tooth movement and endodontic surgery to the planning and placement of dental implants (3). Accurate three-dimensional assessment of the IC's morphology including its dimensions, trajectory, and spatial relationship to adjacent structures is paramount to avoiding iatrogenic complications such as neurovascular injury, hemorrhage, or sensory disturbance (4).

On the other hand, the loss of a maxillary central incisor (MCIL) presents a profound restorative challenge, particularly in the context of implant therapy. Following extraction, the alveolar ridge undergoes inevitable resorptive remodeling, a process that may extend to alter the architecture of the adjacent IC (5, 6). A previous study suggests that the IC is not a static landmark but a dynamic structure susceptible to change following tooth loss. Potential transformations, such as dimensional alterations in canal diameter and length, changes in bucco-palatal orientation, and approximation of its contents to the residual alveolar crest, have significant clinical implications. These changes directly influence the available bone volume for implant placement, modify the risk of perforation during osteotomy, and complicate the pre-surgical assessment for rehabilitation in this aesthetically and functionally sensitive zone (7).

While two-dimensional radiography has historically been used for evaluation, it fails to capture the complex three-dimensional anatomy of the IC. Cone-beam computed tomography (CBCT) has revolutionized pre-surgical imaging by providing high-resolution, sub-millimeter accuracy for visualizing such structures. Furthermore, CBCT enables precise multiplanar reconstruction and quantitative assessment, which are indispensable for detailed morphometric analysis and safe pre-surgical planning (8). Despite this technological advancement, detailed normative references describing IC morphology remain scarce, particularly for specific ethnicities including the Vietnamese population. Furthermore, there is a notable lack of comparative analysis elucidating the anatomical changes of the IC following the MICL within this demographic. This gap impedes the development of tailored surgical guidelines. Therefore, this study seeks to elucidate the anatomical changes of the IC by conducting a comparative CBCT analysis between dentate and MCIL subjects in a Vietnamese sub-population. The null hypothesis of this study was that the three-dimensional morphology and dimensions of the incisive canal would not differ significantly between dentate subjects and those with maxillary central incisor loss.

## 2 Materials and methods

### 2.1 Participants

Inclusion criteria subject includes Vietnamese adults (≥18 years); CBCT scan encompassing the entire anterior maxilla. For Dentate group: CBCT scans presence of both maxillary central incisors with intact crowns and roots; no significant periapical pathology or severe bone loss in the anterior region. For MCIL group: CBCT scans encompass unilateral or bilateral loss of at least one maxillary central incisor (with healed alveolar ridge); absence of active infection or large bone defect at the site.

Exclusion criteria comprised CBCT scans with artifacts in the anterior maxilla; patient with history of trauma, surgery, or congenital craniofacial syndrome affecting the maxilla; presence of large pathological lesions (cysts/tumors) in the incisive region. Data on demographic characteristics (age, gender) and clinical findings were recorded using a medical record form. We fully implemented the principles outlined in the Declaration of Helsinki and the PROCESS checklist in this study (9).

### 2.2 Sample size canculation

The sample size was estimated *a priori* for comparing two means using G*Power software (Version 3.1). A power analysis was performed to determine the minimum number of subjects required to detect a clinically meaningful difference in the primary outcome variable (sagittal IC length) between the Dentate and MCIL groups, thereby ensuring adequate statistical power (1-*β* = 0.80) and reducing the risk of Type II error. Based on a previous study by Bermúdez-Pérez M. et al. (10), the mean sagittal IC length was 10.66 ± 2.35 mm in dentate patients vs. 9.05 ± 2.52 mm in edentulous patients, yielding an effect size (d) of 0.66. To detect this difference with 80% power at a two-sided alpha level of 0.05, a minimum of 38 subjects per group was required. Our initial database search yielded a larger pool of eligible dentate subjects. To maximize statistical power for within-group analyses and general characterization, we included all 100 eligible dentate scans. All 38 eligible MCIL scans meeting the criteria were included, meeting the minimum requirement for the primary comparative analysis.

### 2.3 Standardization of CBCT data

The CBCT scans were obtained using a (CBCT) imaging Dentri-S (HDX Will, Korea) dental x-ray unit, field of view (FOV) of 16.0 × 14.5 cm, 80 kVp, 8 mA, pixel spacing at 0.2 mm, slice thickness at 0.2 mm, with a 3-dimensional voxel size of 0.08 mm, a scanning time of 24 s. The scans were selected from the database and verified if they met the inclusion criteria. The inclusion criteria for radiographs mandated high technical quality and the absence of any artifacts capable of compromising image fidelity or interfering with diagnostic assessment.

To ensure objectivity and minimize selection bias, CBCT scans were randomly selected using a computer-generated randomization algorithm from the anonymized pool of eligible cases that met the inclusion criteria. No identifying information was accessible to the researchers at any point in the selection or analysis process. Based on the CBCT images, the radiographic features of the incisive canal and the maxillary buccal bone plate were systematically collected for further assessment.

### 2.4 Image processing

The CBCT images were evaluated by a single experienced oral and maxillofacial radiologist (with >8 years of experience in CBCT interpretation) to ensure measurement consistency. To assess intra-observer reliability, a random subset of 20 CBCT scans (approximately 15% of the sample) was re-evaluated by the same observer after a washout period of 4 weeks. The intraclass correlation coefficient (ICC) for continuous measurements ranged from 0.89 to 0.97, indicating excellent intra-observer reliability. Kappa coefficients for morphological classifications ranged from 0.85 to 0.92, indicating strong agreement. Inter-observer reliability was not assessed as a single observer performed all measurements, which minimized inter-observer variability; however, this is acknowledged as a limitation.

#### 2.4.1. On the sagittal plane

**Figure 1:**
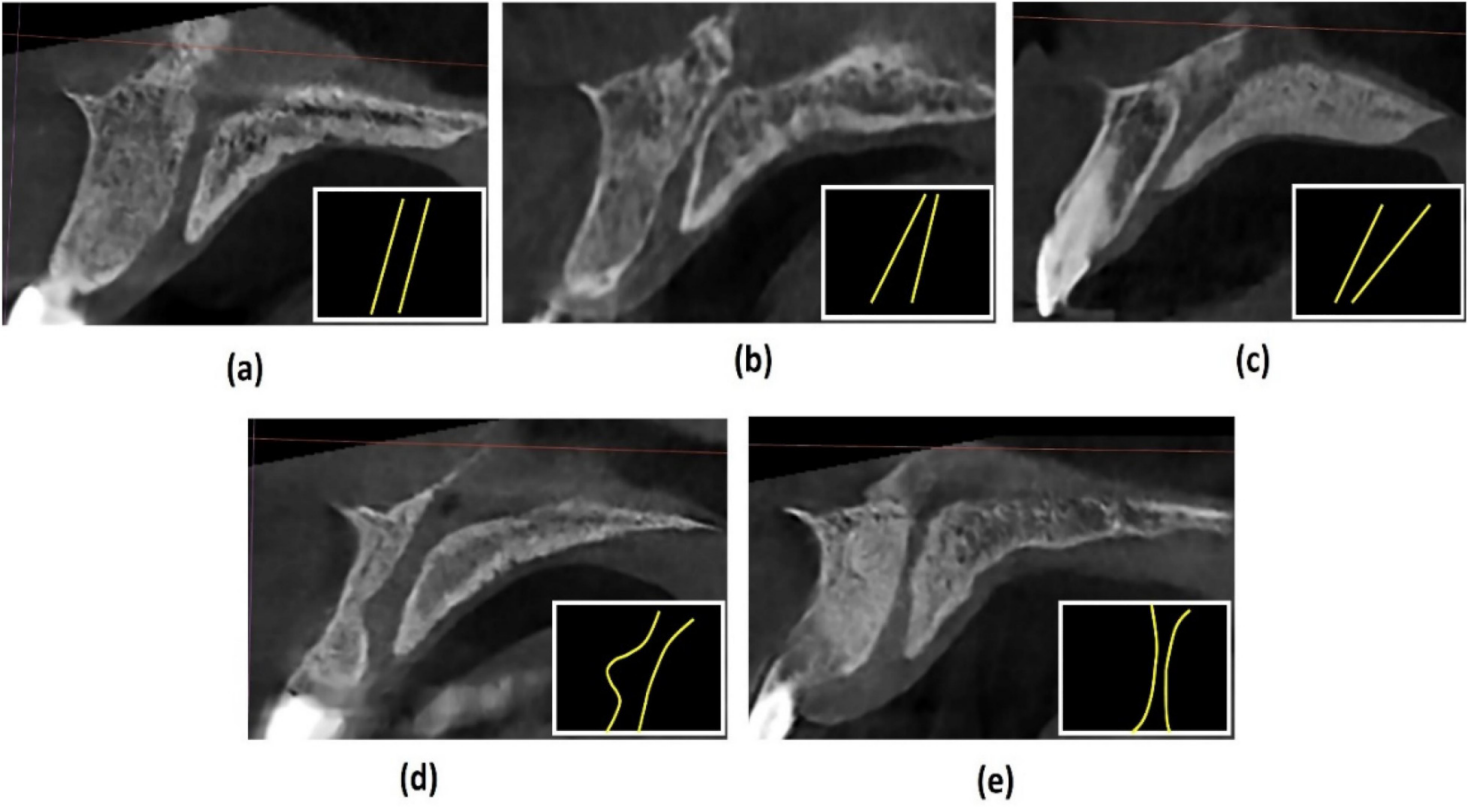
Shape of IC on the sagittal plane. **(a)** Cylindrical shape, **(b)** Funnel shape, **(c)** Inverted funnel shape, **(d)** Spindle shape, **(e)** Hourglass shape. Yellow double-lines in rectangular white box: animation relevant to each morphologic classification of the IC.

The morphologic variations of the canal were classified into five groups, which includes cylindrical, funnel, inverted-funnel, spindle, and hourglass shapes (Figure 1).

**Figure 2:**
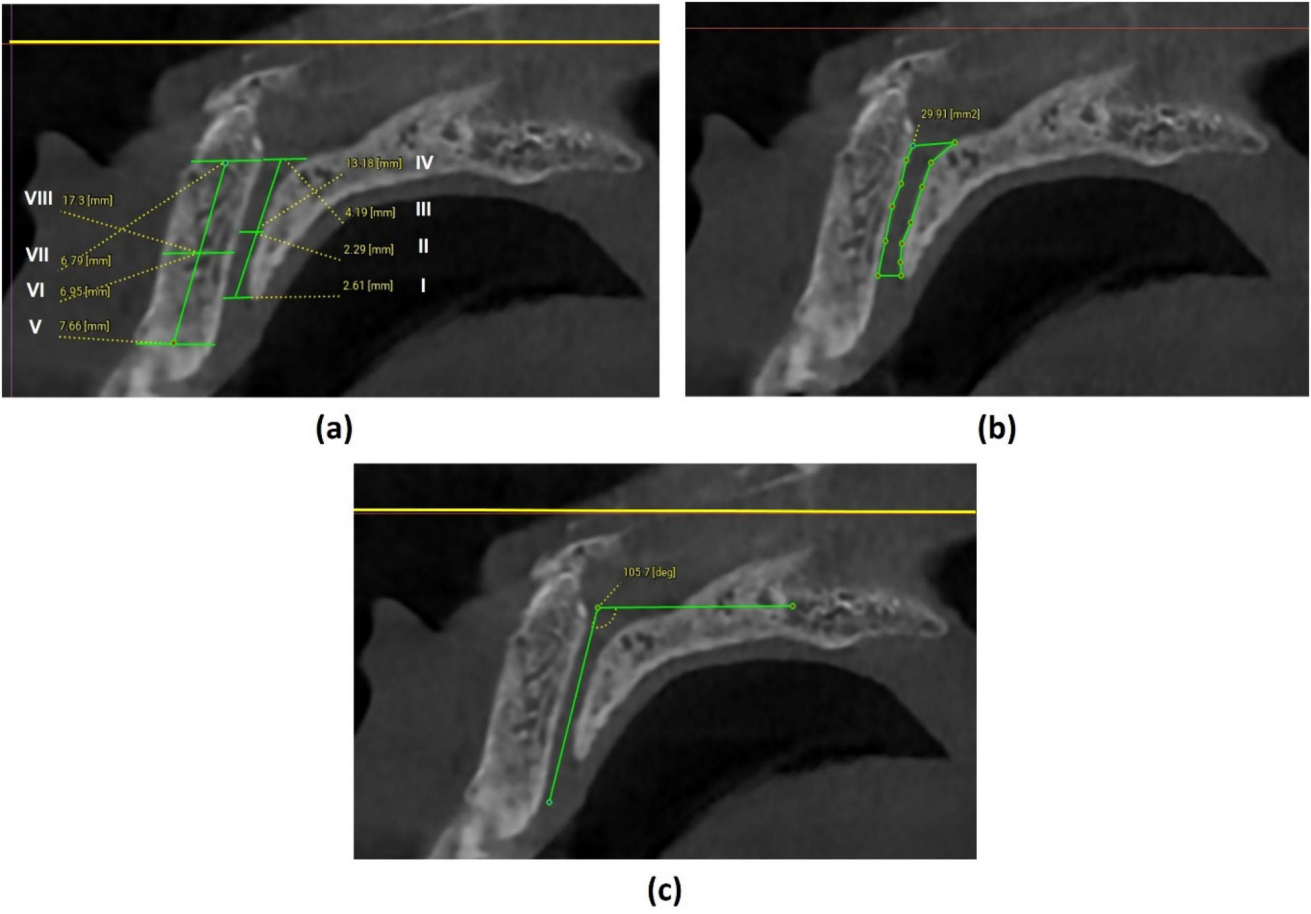
Measurements of IC and the buccal alveolar bone plate on the sagittal plane. **(a)** Diameters and length of the IC and the buccal bone plate at different landmarks: I - diameter of the IC at incisive foramen (mm), II - the diameter of the IC at middle of the IC (mm); III - diameter of the IC at nasal foramen (mm), IV - length of the IC (mm), V - maxillary buccal alveolar bone thickness at crest (mm), VI - maxillary buccal alveolar bone thickness at the middle (mm), VII - maxillary buccal alveolar bone thickness at the nasal spine (mm), VIII - maxillary buccal alveolar bone high (mm); **(b)** Area of the IC (mm^2^); **(c)** Angulation of the IC to the horizontal plane (˚). Yellow lines: the line parallel to the anterior—posterior nasal spine plane).

The dimensions of the IC and the maxillary buccal alveolar bone plate were measured in millimeters following the directional guided planes:

- **(I)** Incisive canal (IC) diameter at palatal foramen: Maximum mesiodistal dimension of the canal at its palatal termination, measured perpendicular to the long axis of the canal.
- **(II)** IC diameter at middle: Measured at the midpoint of the canal length (defined as the point exactly halfway between the palatal and nasal openings along the canal’s central axis).
- **(III)** IC diameter at nasal foramen (nasal opening): Maximum dimension of the canal at its nasal termination, measured perpendicular to the long axis.
- **(IV)** IC length: Linear distance from the center of the palatal opening to the center of the nasal opening along the canal’s central axis.
- **(V)** Buccal alveolar bone plate (BABP) width at crest: Horizontal thickness of the buccal cortical plate measured 1 mm apical to the alveolar crest, perpendicular to the long axis of the central incisor.
- **(VI)** BABP width at middle
- **(VII)** BABP width at nasal spine (Figure 2a)
- **(VIII)** BABP height: Vertical distance from the alveolar crest to the nasal floor measured along the buccal plate.
- IC area: Cross-sectional area of the canal measured at its widest point on sagittal view, using manual planimetry. (Figure 2b)
- IC angulation: Angle between the line connecting the palatal and nasal opening centers and the horizontal plane (anterior-posterior nasal spine plane). (Figure 2c)

#### 2.4.2 On the coronal plane

**Figure 3:**
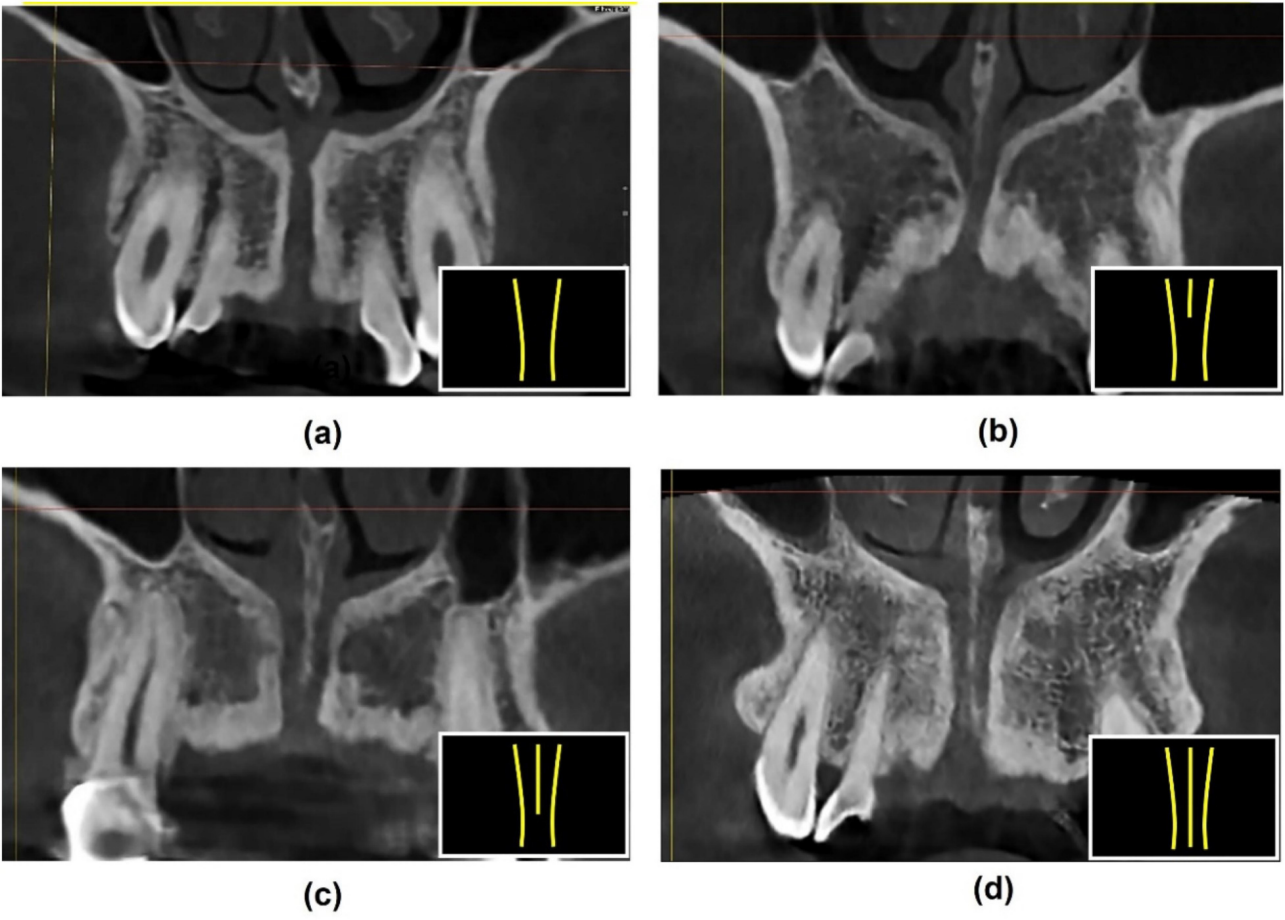
Morphologic variations of the IC on the coronal plane were classified into four groups: **(a)** No division, **(b)** nasal division level, **(c)** middle division level, **(d)** oral division level. Yellow lines in rectangular white box: animation for each morphologic classification of the IC.

The morphologic variations of the canal were classified into four groups: no division, nasal division level, middle division level, and oral division level (Figure 3).

**Figure 4:**
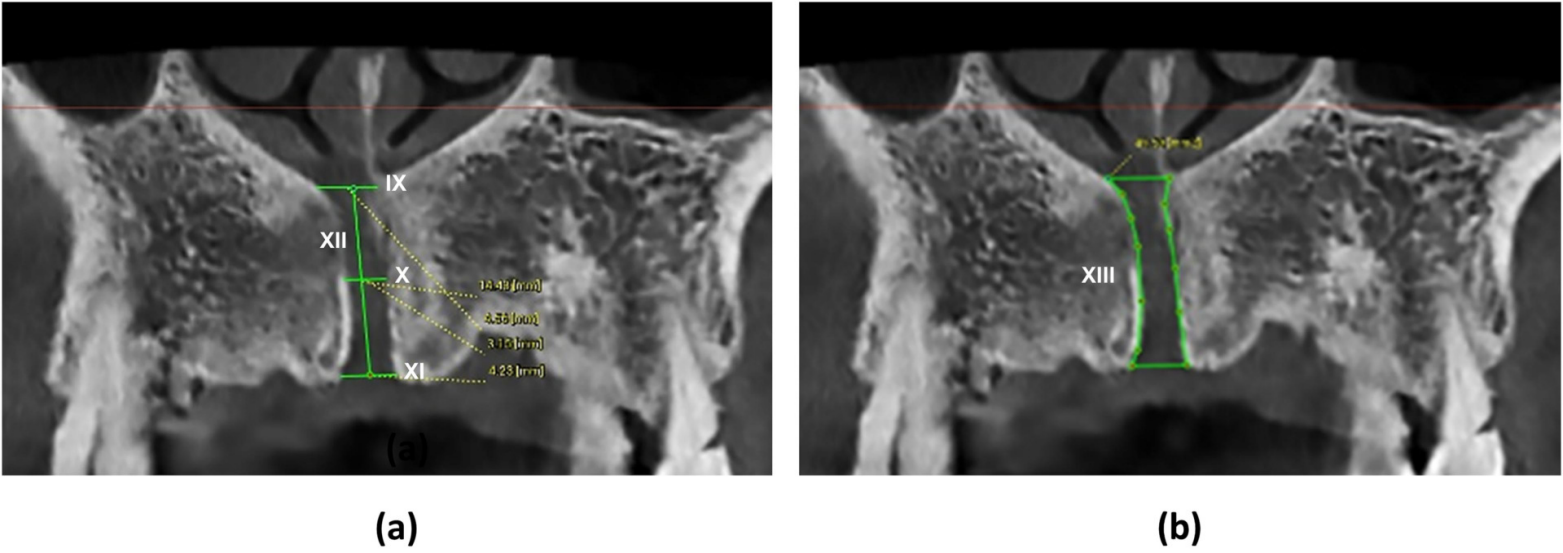
Measurements of the IC were measured in millimeters on the coronal plane. **(a)** Diameters and length of the IC at different landmarks: IX – diameter of the IC at palatal opening, X – diameter of the IC at middle of the IC (mm), XI – diameter of the IC at nasal foramen (mm), XII – length of the IC (mm); **(b)** XIII – Area of the IC (mm^2^). Red lines: the line parallel to the anterior-posterior nasal spine plane.

- **(IX)** IC diameter at the palatal foramen.
- **(X)** IC diameter at middle: Measured at the midpoint of the canal length (proportionally defined as 50% of the distance from palatal to nasal openings).
- **(XI)** IC diameter at nasal foramen: Maximum buccopalatal dimension at the nasal termination.
- **(XII)** IC length: Linear distance from palatal to nasal opening centers in coronal view. (Figure 4a)
- **(XIII)** IC area: Cross-sectional area measured at the midpoint of the canal. (Figure 4b)

The use of proportional midpoints was chosen to account for inter-individual variation in canal length, ensuring that “middle” measurements corresponded to anatomically comparable positions across subjects.

#### 2.4.3 On the axial plane

**Figure 5:**
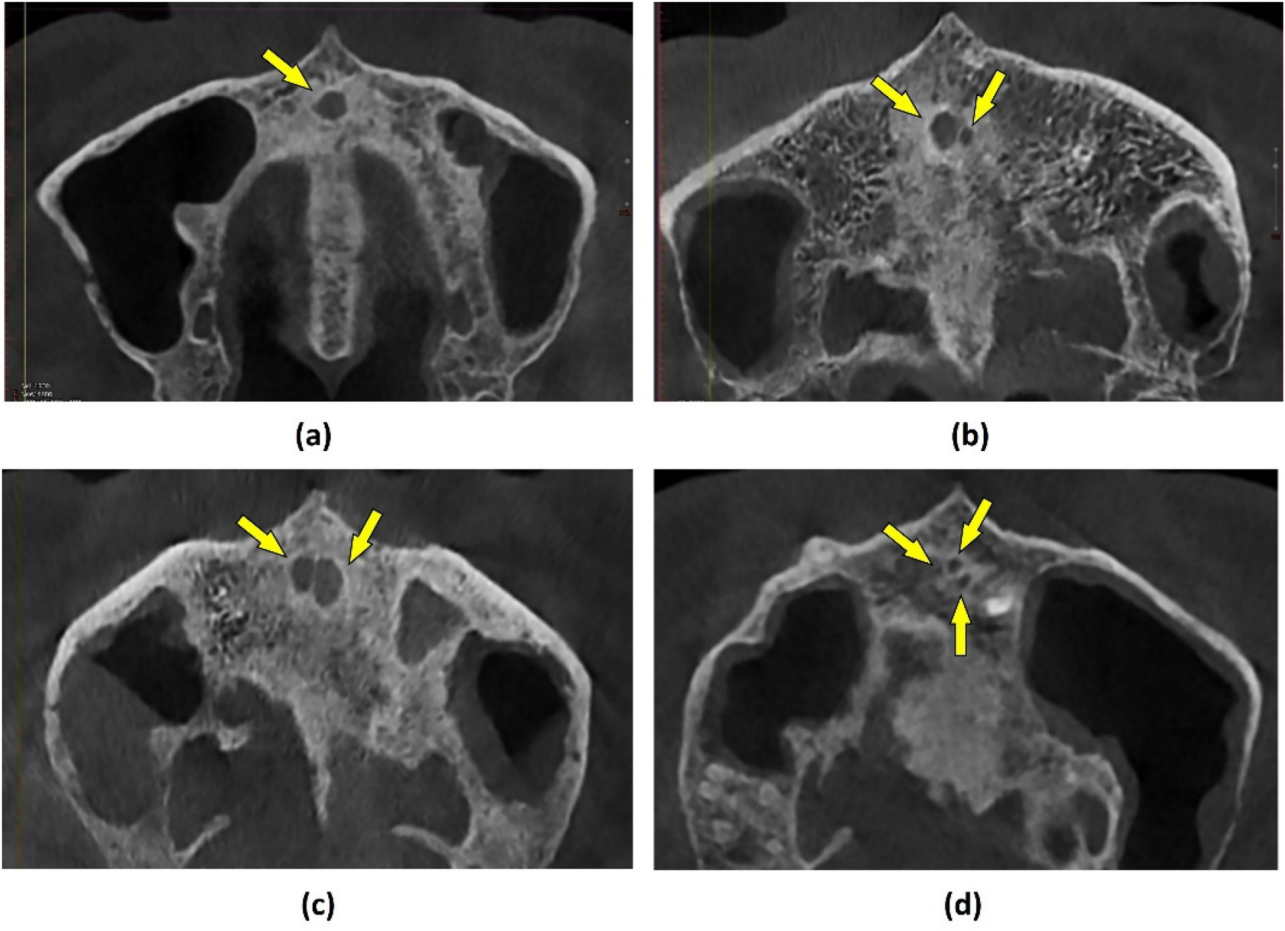
Number of nasal foramen on the axial plane. **(a)** 1 foramen; **(b,c)** 2 foramina; **(d)** 3 foramina. Yellow arrows: position of the nasal foramina.

The number of nasal foramen on the axial plane of the IC were classified into three groups : 1 foramen, 2 foramina, and 3 foramina (Figure 5).

### 2.5 Data processing

Statistical analyses were performed using Microsoft Excel 2016 and SPSS version 26.0. Continuous variables were analyzed using analysis of covariance (ANCOVA) with age as a covariate. Categorical variables were analyzed using logistic regression (binary for dichotomous outcomes, multinomial for polytomous outcomes) with age as an independent variable. Adjusted means and 95% confidence intervals were calculated using the emmeans package. Effect sizes are reported as partial *η*^2^ for ANCOVA and odds ratios for logistic regression. Statistical significance was set at *p* < 0.05.

## 3 Results

### 3.1 Demographic data

A total of 138 subjects were included in this cross-sectional study and divided into two groups: Dentate (*n* = 100) and Maxillary Central Incisor Loss (MCIL, *n* = 38). The demographic characteristics are summarized in Table 1. The mean age (± standard deviation) was significantly higher in the MCIL group (57.5 ± 16.1 years) compared to the Dentate group (36.5 ± 17.9 years) (*p* < 0.01, independent t-test). The overall gender distribution comprised 71 males (51.4%) and 67 females (48.6%). Within the MCIL group, the distribution of tooth loss was as follows: loss of one incisor (50.0%, *n* = 19), two incisors (15.8%, *n* = 6), and more than two incisors (34.2%, *n* = 13).

**Table 1:** Demographic information of the patients.

Parameters	n	% or Mean (± SD)	*P*-value
Age (years)			
Dentate		35.5 ± 17.9	**<0.01** ^a^
MCIL		57.5 ± 16.1	
Gender			
Male	71	51.4	>0.05^a^
Female	67	48.6	
Total	138	100	
Incisors loss situation in the MCIL group			
Only 1 central Incisor was lost	19	50	>0.05^b^
2 central incisors were lost	6	15.8	
More than 2 central incisor were lost	13	34.2	
Total	38	100	

a Independent *t*-test.

b One-way ANOVA.

Significant differences with *p* < 0.05.

The bold values presents the significant difference (*p*-value < 0.05).

### 3.2 Morphological characteristics of the IC

The morphological findings for the incisive canal (IC) are presented in Table 2. On sagittal plane, the most prevalent shape in both groups was cylindrical (Dentate: 52.0%, *n* = 52; MCIL: 57.9%, *n* = 22), followed by funnel shape (Dentate: 6.0%, *n* = 6; MCIL: 15.8%, *n* = 6). The spindle shape was the least common (Dentate: 6.0%, *n* = 6; MCIL: 2.6%, *n* = 1). No statistically significant differences were found between the groups (*p* > 0.05, Chi-square test). On coronal plane, the majority of subjects in both groups presented with no division of the IC (Dentate: 62.0%, *n* = 62; MCIL: 47.4%, *n* = 18). When present, division occurred most frequently at the upper third (nasal level) in both groups. No significant inter-group differences were observed (*p* > 0.05). On axial plane, a single nasopalatine foramen was the most frequent finding in the Dentate group (64.0%, *n* = 64), whereas two foramina were most common in the MCIL group (52.6%, *n* = 20). Three foramina were observed in only one dentate subject. These differences were not statistically significant (*p* > 0.05) (Figure 5).

**Table 2:** Morphology of the IC and number of nasal forramen following tooth loss situation.

Parameter	Category	Dentate (*n* = 100)	MICL (*n* = 38)	*p*-value
Shape on Sagittal Plane				
	Cylindrical	52 (52.0%)	22 (57.9%)	**0**.**046**^a^
	Funnel	6 (6.0%)	6 (15.8%)	
	Inverted funnel	25 (25.0%)	3 (7.9%)	
	Hourglass	11 (11.0%)	6 (15.8%)	
	Spindle	6 (6.0%)	1 (2.6%)	
Shape on Coronal Plane				
	No division	62 (62.0%)	18 (47.4%)	0.103^a^
	Nasal division	22 (22.0%)	15 (39.5%)	
	Middle division	12 (12.0%)	5 (13.2%)	
	Oral division	4 (4.0%)	0 (0.0%)	
Number of Foramina on Axial Plane				
	1 foramen	64 (64.0%)	18 (47.4%)	0.122^a^
	2 foramina	35 (35.0%)	20 (52.6%)	
	3 foramina	1 (1.0%)	0 (0.0%)	

a Chi-Square tests of independence. .

A Fisher's Exact Test confirmed the significant result for the Shape on sagittal plane shape (*p* = 0.039).

MICL, maxillary incisor loss.

The bold values presents the significant difference (*p*-value < 0.05).

### 3.3 Morphometric changes associated with gender

Comparative analysis between males and females across the entire sample revealed several statistically significant differences (*p* < 0.05, independent t-test), as detailed in Table 3. Males exhibited larger dimensions than females for most parameters. In terms of the IC, males had significantly greater sagittal diameters at the palatal opening (3.67 ± 0.98 mm vs. 2.75 ± 0.76 mm), middle (2.84 ± 0.94 mm vs. 2.73 ± 0.85 mm), and nasal opening (4.73 ± 2.21 mm vs. 4.33 ± 2.07 mm). The sagittal IC area was also larger in males (34.92 ± 10.32 mm^2^ vs. 27.52 ± 10.13 mm^2^). As for the BABP, all buccal bone width measurements were significantly larger in males: crestal width (7.52 ± 1.40 mm vs. 7.12 ± 1.59 mm), middle width (7.70 ± 1.36 mm vs. 6.77 ± 1.67 mm), and nasal width (10.41 ± 2.28 mm vs. 9.79 ± 1.69 mm). Buccal bone height did not differ significantly between genders.

**Table 3:** Quantitative parameters of the IC and the buccal alveolar bone plate following gender.

Parameters	Male (Mean ± SD)	Female (Mean ± SD)	*p*-value
At sagittal plane			
Canal length (mm)	12.91 ± 2.21	9.81 ± 1.96	**3.48509E-09**
Canal diameter (mm)			
Incisive foramen	3.67 ± 0.98	2.75 ± 0.76	**0**.**0000**
Middle	2.84 ± 0.94	2.73 ± 0.85	0.53
Nasal foramen	4.73 ± 2.21	4.33 ± 2.07	**0**.**027**
Canal area (mm^2^)	34.92 ± 10.32	27.52 ± 10.13	**1.8E-07**
Canal angulation (˚)	112.10 ± 7.19	112.30 ± 5.89	0.31
Buccal alveolar bone height (mm)	15.21 ± 2.30	14.92 ± 2.64	0.06
Buccal alveolar bone thickness (mm)			
Crest	7.52 ± 1.40	7.12 ± 1.59	0.3
Middle	7.7 ± 1.36	6.77 ± 1.67	**0**.**0004**
Nasal spine	10.41 ± 2.28	9.79 ± 1.69	0.084
At coronal plane			
Canal length (mm)	15.47 ± 2.49	12.72 ± 2.29	**0**.**00001**
Canal diameter (mm)			
Incisive foramen (IF)	4.54 ± 1.04	4.24 ± 0.92	**0**.**01**
Middle	3.40 ± 1.11	3.19 ± 1.22	0.77
Nasal foramen (NF)	4.91 ± 1.51	4.57 ± 1.48	0.62
Canal area (mm^2^)	58.66 ± 14.30	44.99 ± 19.12	**0**.**00066**

Independent *t*-tests. Significant differences with *p* < 0.05.

The bold values presents the significant difference (*p*-value < 0.05).

### 3.4 Morphometric changes associated with MCIL

Comparative analysis between the Dentate and MCIL groups demonstrated specific morphometric alterations associated with tooth loss, summarized in Table 4. In terms of the IC, no significant differences were found between groups for any IC parameter, including sagittal length (Dentate: 11.46 ± 2.48 mm vs. MCIL: 11.75 ± 2.30 mm; *p* > 0.05), diameters, angulation, area, or coronal length. In contrast, all width dimensions of the buccal bone plate were significantly reduced in the MCIL group. The crestal width decreased from 7.36 ± 1.56 mm (Dentate) to 4.50 ± 1.50 mm (MCIL) (*p* < 0.00001). The middle width decreased from 7.35 ± 1.62 mm to 6.03 ± 2.02 mm (*p* < 0.001). The nasal width also showed a significant reduction from 9.68 ± 2.55 mm to 10.06 ± 2.10 mm (*p* < 0.05). Buccal bone height was not significantly different between groups.

**Table 4:** Quantitative parameters of the IC and the buccal alveolar bone plate following tooth loss situation.

Parameters	Dentate (Mean ± SD)	MCIL (Mean ± SD)	*p*-value
At sagittal plane			
Canal length (mm)	11.46 ± 2.48	11.75 ± 2.30	0.15
Canal diameter (mm)			
Incisive foramen	3.54 ± 0.88	3.54 ± 1.13	**0**.**02**
Middle	2.70 ± 0.93	2.74 ± 1.33	0.25
Nasal foramen	4.59 ± 2.18	4.22 ± 2.56	0.43
Canal area (mm^2^)	31.38 ± 11.70	35.40 ± 11.63	0.81
Canal angulation (˚)	112.20 ± 6.56	112.60 ± 6.31	0.51
Buccal alveolar bone height (mm)	15.05 ± 2.52	15.10 ± 3.11	0.13
Buccal alveolar bone thickness (mm)			
Crest	7.36 ± 1.56	4.50 ± 1.50	**0**.**000001**
Middle	7.35 ± 1.62	6.03 ± 2.02	**0**.**000**
Nasal spine	9.86 ± 2.14	10.06 ± 2.10	0.64
At coronal plane			
Canal length (mm)	14.45 ± 2.72	13.72 ± 2.60	0.27
Canal diameter (mm)			
Incisive foramen (IF)	4.41 ± 1.01	4.19 ± 1.54	0.99
Middle	3.28 ± 1.01	3.52 ± 0.94	0.68
Nasal foramen (NF)	4.84 ± 1.17	4.53 ± 0.94	0.28
Canal area (mm^2^)	55.42 ± 17.89	51.27 ± 17.16	0.46

Independent *t*-tests. Significant differences with *p* < 0.05.

MICL, Maxillary Incisor Loss.

The bold values presents the significant difference (*p*-value < 0.05).

After adjustment for age, buccal alveolar bone plate dimensions remained significantly reduced in the MCIL group at all measurement levels [crestal: F(1,134) = 42.15, *p* < 0.001, partial *η*^2^ = .284; middle: F(1,134) = 16.89, *p* < 0.001, partial *η*^2^ = .143; nasal: F(1,134) = 8.76, *p* = 0.009, partial *η*^2^ = 0.089]. The adjusted mean difference in crestal width was −2.86 mm (95% CI: −3.65 to −2.07). In contrast, no incisive canal parameters showed statistically significant differences between groups after age adjustment (*p* > 0.05). Logistic regression with age as a covariate revealed no significant association between tooth loss status and morphological classification of the incisive canal (all *p* > 0.05).

## 4 Discussion

This CBCT-based study provides a detailed three-dimensional analysis of the anatomical relationship between the IC, the buccal alveolar bone plate (BABP), and maxillary central incisor loss (MCIL) in a Vietnamese population. The findings of this study support the acceptance of the null hypothesis, confirming the morphological stability of the incisive canal following the loss of a maxillary central incisor. The principal finding reveals a distinct pattern of post-extraction remodeling: while the BABP undergoes significant dimensional reduction, the IC maintains its morphological and morphometric stability, including its sagittal length.

The most critical finding for clinical implantology is the significant reduction in the width of the BABP at all measured levels following tooth loss. This aligns unequivocally with the established pattern of post-extraction alveolar ridge resorption, where the buccal cortical plate is particularly vulnerable to rapid atrophy (11, 12). This resorption drastically reduces the horizontal bone volume, elevating the risk of buccal plate perforation during implant placement (13, 14).

A pivotal and somewhat unexpected result was the lack of significant change in the sagittal length of the IC following MCIL. This finding contrasts with some previous literature suggesting canal shortening in edentulous areas, but aligns with other studies reporting relative stability (15, 16). This discrepancy may be explained by several interrelated factors specific to our study cohort and the nature of the IC itself. Firstly, the IC is an anatomical conduit within the palatal bone, primarily bordered by the nasal floor superiorly and the palatine process inferiorly. These boundaries are not directly dependent on the periodontal attachment apparatus of the adjacent teeth. Therefore, the physiologic remodeling following extraction, which predominantly affects the alveolar process housing the tooth socket, may have a limited and delayed effect on the osseous structure of the canal proper (17). Secondly, the timeframe from tooth loss to CBCT scan in our MCIL group (implied by “healed alveolar ridge” in inclusion criteria) may not have been sufficient to trigger observable osseous changes in the canal walls. Significant morphological alteration of the IC might require longer-term, advanced resorption of the entire anterior maxilla, potentially spanning years or decades, rather than the initial healing period (18). This cross-sectional design, without precise data on the duration of edentulism, cannot confirm this temporal relationship, but it proposes a hypothesis where the IC exhibits a higher “remodeling threshold” than the adjacent alveolar bone (19, 20).

The stability of the IC's dimensions is a clinically reassuring finding. As noted, the MCIL group was significantly older than the Dentate group. However, the ANCOVA results **(**Table 5**)** confirm that after controlling for age as a covariate, the significant reductions in buccal alveolar bone plate dimensions (crestal, middle, and nasal widths) in the MCIL group persisted, with moderate to large effect sizes (partial *η*^2^ ranging from 0.089 - 0.284). This indicates that the observed bone resorption is independently associated with tooth loss status, not merely a function of the age difference between groups. Conversely, after age adjustment, none of the incisive canal parameters showed statistically significant differences between dentate and MCIL subjects. This reinforces the conclusion that the IC's morphology and dimensions remain stable despite incisor loss and irrespective of patient age, at least within the timeframe represented in this cohort. It suggests that the canal provides a consistent and predictable anatomical landmark for surgical navigation in the anterior maxilla, regardless of incisor presence. This consistency is valuable for planning safe distances for implant placement and osteotomy depth. While the canal's osseous dimensions may not change, the functional proximity of the neurovascular bundle to the surgical field may increase due to the concomitant loss of the overlying alveolar bone, necessitating continued caution (21).

**Table 5:** ANCOVA results for continuous variables with age-adjusted.

Variable	Adjusted mean (dentate)	Adjusted mean (MCIL)	Mean difference	F	*P*-value	Partial *η*²
Buccal alveolar bone plate						
Crestal width	7.36 mm	4.50 mm	−2.86 mm	42.15	**<0.001**	0.284
Middle width	7.35 mm	6.03 mm	−1.32 mm	16.89	**<0.001**	0.143
Nasal width	9.68 mm	8.58 mm	−1.10 mm	8.76	**0**.**009**	0.089
Incisive canal on sagittal plane						
Length	11.46 mm	11.75 mm	+0.29 mm	0.66	0.421	0.007
Palatal diameter	3.67 mm	3.42 mm	−0.25 mm	0.79	0.378	0.008
Middle diameter	2.84 mm	2.79 mm	−0.05 mm	0.02	0.892	<0.001
Nasal diameter	4.73 mm	4.52 mm	−0.21 mm	0.33	0.567	0.004
Area	34.92 mm²	33.45 mm²	−1.47 mm²	0.23	0.634	0.002
Angulation	113.5°	114.2°	+0.7°	0.14	0.712	0.001

ANCOVA test for continuous variables. Significant differences with *p* < 0.05.

MICL, maxillary incisor loss.

The bold values presents the significant difference (*p*-value < 0.05).

On the other hand, the observed morphological patterns, with the cylindrical shape being predominant, are consistent with reports from other ethnic populations (22, 23). The absence of significant morphological shift between groups further supports the concept of the IC's structural stability. The significant sexual dimorphism identified, with males presenting larger IC and BABP dimensions, is congruent with general craniofacial anatomical differences (24). In a recent Vietnamese study by Phạm et al. (2025) evaluated IC morphology in 145 dentate Vietnamese adults using CBCT, providing a valuable population-specific comparison (25). The mean IC length reported in that study (11.42 ± 2.21 mm) is nearly identical to our finding of 11.46 ± 2.48 mm, and both studies identified significant sexual dimorphism (male length ∼12.3 mm vs. female ∼10.8 mm). The distribution of coronal plane morphologies was also remarkably consistent: single canal (57.9%), Y-shaped (40.0%), and double canal (2.1%) in both studies. However, sagittal plane shape prevalence differed: we observed cylindrical shape in 52.0% vs. 38.6% in Phạm et al., while funnel shape was 6.0% in our study vs. 23.4% in theirs. This discrepancy likely reflects differences in morphological classification systems rather than true population variation. Buccal bone plate thickness (7.36–7.97 mm) and angulation (112–113°) were comparable. Overall, Vietnamese IC normative values for length and coronal morphology are consistent with other Asian populations (Turkish, Palestinian, Japanese), while shape classification variability highlights the need for standardized criteria (15, 16, 22, 23). Importantly, the close agreement between two independent Vietnamese studies supports the reliability of our findings and confirms that IC dimensions in Vietnamese individuals fall within the range reported globally, suggesting that international surgical guidelines are applicable to this population with the caveat that individual CBCT assessment remains essential.

The finding that maxillary central incisor loss is associated with significant horizontal resorption of the BABP but does not significantly alter the morphology or dimensions of the incisive canal carries several important clinical implications. First, the reduction in buccal bone width, which was observed most dramatically at the crestal level (from 7.36 mm to 4.50 mm) indicates that the residual ridge may frequently be insufficient to accommodate standard-diameter implants (typically 3.5–4.5 mm) without concomitant bone augmentation procedures. Clinicians should anticipate the need for guided bone regeneration or the use of narrower-diameter implants in MCIL patients. Second, because the IC dimensions remain stable, the canal continues to serve as a reliable anatomical landmark for preoperative planning. The mean sagittal IC length of approximately 11.5 mm (range: 9.2–14.2 mm) suggests that osteotomy depths exceeding 10 mm in the midline region carry a risk of incisive canal penetration, particularly in shorter canals. Third, the preserved IC architecture implies that the nasopalatine neurovascular bundle retains its original position relative to palatal bone, even as the buccal plate resorbs. This creates an asymmetric bone deficiency that may shift the ideal implant axis toward a more palatal emergence profile to avoid canal violation. Fourth, the sexual dimorphism observed (males having larger dimensions) suggests that female patients may have narrower safety margins and may more frequently require pre-implant augmentation. Taken together, these findings underscore the necessity of CBCT-based preoperative assessment to simultaneously evaluate the diminished buccal bone volume requiring augmentation and the stable IC landmark to prevent iatrogenic injury.

This study has several limitations. Its cross-sectional design cannot establish causality or trace the timeline of anatomical changes. The significant age difference between groups is a confounding factor, as age-related changes could independently influence dimensions. Crucially, the lack of precise data on the duration since tooth loss for each MCIL subject limits our ability to correlate morphological findings with the timeframe of edentulism. Consequently, our conclusions regarding IC stability are limited to the short-to-intermediate post-extraction period. We cannot exclude the possibility that long-term edentulism that might eventually induce measurable morphological changes in the IC. While intra-observer reliability was excellent, inter-observer reliability was not assessed as all measurements were performed by a single experienced observer. Furthermore, the sample was drawn from a specific Vietnamese sub-population, which may affect generalizability. Future longitudinal studies with documented extraction timelines and incorporated multiple observers are needed to elucidate the potential long-term effects of tooth loss on the IC and to evaluate the generalizability.

## 5 Conclusion

In conclusion, this study demonstrates that maxillary central incisor loss precipitates significant horizontal resorption of the buccal alveolar bone plate, while the incisive canal, including its length, remains a morphometrically stable structure in the short to medium term following tooth loss. This underscores the necessity of CBCT for pre-surgical planning: to accurately assess the diminished buccal bone volume requiring augmentation, while utilizing the stable architecture of the IC as a reliable landmark to avoid iatrogenic injury during implant therapy in the aesthetic zone.

## Data Availability

The raw data supporting the conclusions of this article will be made available by the authors upon request, due to privacy/ethical restrictions.
